# Role of AMP-Activated Protein Kinase on Steroid Hormone Biosynthesis in Adrenal NCI-H295R Cells

**DOI:** 10.1371/journal.pone.0030956

**Published:** 2012-01-25

**Authors:** Andrea Hirsch, Dagmar Hahn, Petra Kempná, Gaby Hofer, Primus E. Mullis, Jean-Marc Nuoffer, Christa E. Flück

**Affiliations:** 1 Division of Pediatric Endocrinology, Diabetology and Metabolism, Department of Pediatrics, Inselspital, University Hospital, University of Bern, Bern, Switzerland; 2 Department of Clinical Research, Inselspital, University Hospital, University of Bern, Bern, Switzerland; 3 Institute of Clinical Chemistry, Inselspital, University Hospital, University of Bern, Bern, Switzerland; Florida International University, United States of America

## Abstract

Regulation of human androgen biosynthesis is poorly understood. However, detailed knowledge is needed to eventually solve disorders with androgen dysbalance. We showed that starvation growth conditions shift steroidogenesis of human adrenal NCI-H295R cells towards androgen production attributable to decreased HSD3B2 expression and activity and increased CYP17A1 phosphorylation and 17,20-lyase activity. Generally, starvation induces stress and energy deprivation that need to be counteracted to maintain proper cell functions. AMP-activated protein kinase (AMPK) is a master energy sensor that regulates cellular energy balance. AMPK regulates steroidogenesis in the gonad. Therefore, we investigated whether AMPK is also a regulator of adrenal steroidogenesis. We hypothesized that starvation uses AMPK signaling to enhance androgen production in NCI-H295R cells. We found that AMPK subunits are expressed in NCI-H295 cells, normal adrenal tissue and human as well as pig ovary cells. Starvation growth conditions decreased phosphorylation, but not activity of AMPK in NCI-H295 cells. In contrast, the AMPK activator 5-aminoimidazole-4-carboxamide (AICAR) increased AMPKα phosphorylation and increased CYP17A1-17,20 lyase activity. Compound C (an AMPK inhibitor), directly inhibited CYP17A1 activities and can therefore not be used for AMPK signaling studies in steroidogenesis. HSD3B2 activity was neither altered by AICAR nor compound C. Starvation did not affect mitochondrial respiratory chain function in NCI-H295R cells suggesting that there is no indirect energy effect on AMPK through this avenue. In summary, starvation-mediated increase of androgen production in NCI-H295 cells does not seem to be mediated by AMPK signaling. But AMPK activation can enhance androgen production through a specific increase in CYP17A1-17,20 lyase activity.

## Introduction

The human adrenal cortex produces mineralocorticoids, glucocorticoids and androgens; the latter are also synthesized in the gonads of both sexes. Several common enzymes are involved in the strictly regulated biosynthesis of androgens from cholesterol in the adrenals and the gonads [1]. However, the detailed regulation which is tissue specific, developmentally determined and rhythmic remains unknown. Key enzymes for human androgen biosynthesis in the zona reticularis of the adrenal cortex and the gonads include CYP17A1 (17α-hydroxylase/17,20 lyase) and HSD3B2 (3β-hydroxysteroid dehydrogenase type II (3βHSD)). These genes/enzymes are tightly regulated to foster androgen production from the zona reticularis of the adrenal cortex during adrenarche [2]. Although the signaling network underlying adrenarche is still unknown, some of the events have been described and include enhanced CYP17-17,20 lyase activity and decreased HSD3B2 activity [2].

In previous study we showed that serum-free (starvation) growth conditions shift steroidogenesis of human adrenal NCI-H295R cells towards androgen production [3]. This shift was mainly attributed to an increase in CYP17-17,20 lyase activity due to enhanced serine phosphorylation of CYP17 and a decrease of HSD3B2 expression and activity [3]. Serum-free, starvation growth conditions cause cellular stress. Stress stimulates androgen production through ACTH activating the cAMP and PKA pathway, which then modulate gene expression and phosphorylation of different steroidogenic proteins [4]. Most types of stress (e.g. glucose deprivation, metabolic poisons) influence the intracellular energy transfer that is mediated by the nucleotide adenosine-5′-triphosphat (ATP) [5]. It is of critical importance for the cell to maintain the cellular AMP∶ATP ratio, even in response to metabolic stress. AMP-activated protein kinase (AMPK) is certainly one of the best-known sensors perceiving changes in cellular energy [6]. In response to metabolic stress that depletes ATP, AMPK switches off ATP-consuming metabolic pathways and switches on catabolic processes to provide ATP [5], [7], [8].

AMPK belongs to a family of serine/threonine protein kinases and forms a heterotrimeric complex which consists of a catalytic α subunit and two regulatory subunits, β and γ. In mammals, two or three isoforms of each subunit, encoded by distinct genes, may form at least 12 different heterotrimers and the expression pattern of these heterotrimers differs among various tissues [9], [10], [11]. AMPK activity is regulated by the following three known mechanisms. First, if AMP is increased intracellular after energy consumption, AMP binds allosterically to a regulatory γ subunit of AMPK and induces a conformational change that allows the activation of AMPK by phosphorylation [12]. Second, the constitutively active tumor suppressor LKB1 is the main upstream kinase and may activate an α subunit of AMPK by phosphorylating Thr172 [6], [13], [14], [15]. Third, protein phosphatases such as PP2Cα dephosphorylate and inactivate AMPK [12]. These phosphatases may also be inhibited by AMP [12].

Previous studies established a regulatory role of AMPK of steroidogenesis of the gonad [16], [17]. For instance, AICAR-mediated AMPK phosphorylation/activation was found to decrease progesterone secretion by way of ERK1/2 signaling pathway in rat granulosa cells [17]. Therefore, in this study, we determined whether AMPK is also a regulator of adrenal steroidogenesis. Since starvation clearly enhances androgen production in adrenal NCI-H295R cells, we postulate that this might be so using the signaling network of AMPK.

## Materials and Methods

### Ethics Statement

Normal human ovary and adrenal tissue samples were obtained from institutional tissue banks owned by Dres. Michael Müller and George Thalmann, Inselspital Bern, Switzerland and approved by the institutional review board of the Inselspital Bern, Bern as well as the “Kantonale Ethikkommission Bern”, Bern, Switzerland (www.kek-bern.ch). Pig ovarian tissue was provided by the regional slaughterhouse (Micarna SA, Courtepin, Switzerland; www.micarna.ch).

### Materials

The antibodies against (phospho-)AMPKα and (phospho-)acetyl-CoA carboxylase (ACC) were purchased from Cell Signaling Technology (AMPK and ACC Antibody Sampler Kit #9957, Danvers, MA, USA), β-actin and anti-HA antibodies were received from Sigma (Sigma-Aldrich, Buchs, Switzerland). Goat anti-rabbit and goat anti-mouse horseradish peroxidase-conjugated antibody was obtained from Santa Cruz Biotechnology (Santa Cruz, CA, USA). Rabbit polyclonal antibody detecting human CYP17A1 was custom made by Genscript (Genscript, Piscataway, NJ) [3]. Radioactive-labeled (7(N)-3H)pregnenolone (NET-039) and (1,2,6,7(N)-3H)DHEA (NET-814) were procured from Perkin Elmer (Boston, MA, USA), and (1,2,6,7(N)-3H)17α-hydroxypregnenolone hydroxypregnenolone (ART-1233) and (4-14C)progesterone (ART-1398) were purchased from American Radiolabeled Chemicals (St. Louis, MO, USA). Trilostane was extracted in absolute ethanol from tablets commercially available as Modrenal (Bioenvision, New York, NY, USA). Wild-type HA-AMPK or HA-AMPK-DN constructs of subunits alpha 1 and 2 were generously provided by Ken Inoki [18]. DNA templates for short hairpin RNA of AMPKα1 or AMPKα2 were cloned into a pSUPER vector resistant against puromycin (kindly provided by Christoph Schild, Bern). Inhibitors, activators and all other chemicals for respirometric measurements were of standard analytical grade and were purchased from Sigma or Merck (Darmstadt, Germany).

### RNA Isolation and RT-PCR

Total RNA from NCI-H295A and H295R cells, pig and human primary ovarian cell cultures as well as from total human adrenal tissues was isolated using the TRIzol method according to the manufacturer's instructions (Invitrogen, Paisley, UK). RNA was reverse-transcribed to cDNA using the Improm RNA Transcriptase kit (Promega, Madison, WI, USA) and 0.5 µg random primer (Promega) per 1 µg of RNA at 42°C for 1 h. For the semiquantitative RT-PCR, cDNA (100 ng) was amplified by Go-TAQ polymerase (Promega) and specific primers (sequences available on request) in a final volume of 25 µl. PCR conditions were as follows: 45 sec at 95°C, 45 sec at 54–60°C, 45 sec at 72°C for 30 cycles. PCR products were separated by electrophoresis on 1.5% agarose gels, visualized by ethidium bromide staining and detected on an Alpha Imager 3400 (Alpha Innotech, San Leandro, CA, USA). For quantitative Real-Time PCR (qRT-PCR), the 7500 Fast Real-Time PCR System (Applied Biosystems, Foster City, CA, USA) was used. In brief, PCR reactions were performed in 96-well plates (MicroAmp, Applied Biosystems) using cDNA prepared as described above. We used ABsolute QPCR SYBR Green Mix (ABgene, Thermo Fisher Scientific, Wohlen, Switzerland), 1 µl (20 pmol/µl) specific primers (Microsynth, Balgach, Switzerland) and 50 ng cDNA in a total volume of 25 µl. Relative expression values were determined by the 2-ΔΔCt method using 18S rRNA as the reference gene. Amplification curves and the mean Ct values were calculated using the 7500 Fast System SDS software (Applied Biosystems).

### Cell Culture and Treatment

Human adrenal NCI-H295R cells were from American Type Culture Collection (Manassas, VA, USA). Cells were cultured under standard conditions (growth medium) in Dulbecco's modified Eagle's/Ham's F-12 medium containing L-glutamine and 15 mM HEPES (GIBCO, Paisley, UK) supplemented with 5% NuI serum, 0.1% selenium/insulin/transferrin, penicillin (100 U/ml; GIBCO), and streptomycin (100 µg/ml; GIBCO). The serum-free NCI-H295R medium contained Dulbecco's modified Eagle's/Ham's F-12 medium as well as penicillin (100 U/ml; Life Technologies, Paisley, UK), and streptomycin (100 µg/ml; Life Technologies). For RNA and protein extraction experiments and for steroid labeling experiments, cells were grown in growth medium in 6-well plates. After subculturing for 24 hours, medium was replaced, and cells were treated in serum-free medium for 48 h unless indicated differently. AICAR (Sigma-Aldrich) was dissolved in serum-free NCI-H295R medium at stock concentrations of 10 mM (final concentration: 1 mM) and compound C (Calbiochem, Darmstadt, Germany) was dissolved in DMSO at stock concentrations of 10 mM. NCI-H295R cells were treated with 1 mM AICAR or compound C at indicated concentrations. Control cells were treated with 0.2% (v/v) DMSO or medium.

### Steroid Labeling

Steroid metabolism was labeled by adding either 100,000 cpm (^3^H) pregnenolone, (^3^H) DHEA or (^3^H) 17α-hydroxypregnenolone for 90 min. Steroids were extracted from medium as previously described [19] and separated on thin layer chromatography (TLC) plates (Macherey-Nagel, Düren, Germany). For specific analysis of the CYP17 activities, cells were treated with 1 µM trilostane (a specific blocker of HSD3B2 activity) for 90 min before adding labeled steroids. The steroids were visualized on a Fuji PhosphoImager FLA-7000 (Fujifilm, Dielsdorf, Germany) and densitometrically quantified using Multi Gauge software (Fujifilm). Steroid conversion was calculated as percentage of radioactivity incorporated in a specific steroid hot spot compared to total radioactivity added to the reaction.

### Protein Extraction and Western Blot Analysis

In brief, cells were treated as described above, washed with ice-cold PBS and harvest in 250 µl lysis buffer (200 mM Tris-HCl, pH 7.5, 150 mM NaCl, 1 mM EDTA, 1% Triton X-100, protease and/or phosphatase inhibitors). Lysates were passed three times through a 25 G syringe, centrifuged at 13,000×g for 10 min at 4°C and supernatants were collected. Protein concentration was measured using *DC* Protein Assay (Bio-Rad, Hercules, CA, USA). SDS loading buffer (62.5 mM Tris–HCl, pH 6.8; 2% sodium dodecylsulfate, 10% glycerol, 100 mM dithiotreitol, 0.01% bromophenol blue) was mixed with 20 µg of total cell lysates, heated for 5 min at 95°C, separated on a 10% SDS-PAGE gel and blotted on Immobilon P transfer membrane (Millipore, Bedford, MA, USA) using the semi-dry transfer method. Blocking and staining with antibodies was performed according to the manufacturer's recommendations (Cell Signaling Technology or Sigma-Aldrich), For CYP17 detection, membranes were blocked with 5% non-fat dry milk and the secondary antibody (1∶5000) was dissolved in 1× TTBS with 5% BSA.

Protein bands were visualized by ECL Plus substrate reagent (PerkinElmer) and exposed on HyperFilmMP films (GE Healthcare, Fairfield, USA). As control for equal loading, membranes were stripped using 0.2 M NaOH for 30 min, washed and restained against total AMPKα or β-actin.

### Cell Proliferation Assay

The Cell Titer 96 aqueous non-radioactive cell proliferation assay (Promega) was used to determine cell viability and proliferation of NCI-H295R cells grown in different grow conditions or treated with AICAR or compound C. Briefly, cells were cultured on 96-well plates at a density of 20,000 cells/well. The medium was changed to growth medium or starvation medium and cells were allowed to grow for 24 h. Cells were then treated in duplicates with either 1 mM AICAR or 0–20 µM compound C in serum-free medium. After 0, 6, 24 and 48 h cell proliferation was assessed by adding 20 µl of MTS/PMS (3-(4,5-dimethylthiazol-2-yl)-5-(3-carboxymethoxyphenyl)-2-(4-sulfophenyl)-2H-tetrazolium)/(phenazine methosulfate) to the culture medium for 3 hours before reading the absorbance at 490 nm (microplate reader Sunrise Tecan, Salzburg, Austria). In this assay, the absorbance at 490 nm correlates directly to the number of viable cells.

### Microsome Assays

Microsomes containing human recombinant CYP17A1/POR, CYP21A2/POR or HSD3B2 were produced in yeast as previously described [20]. Kinetic assays of CYP17A1, HSD3B2 and CYP21A2 were performed with 30 µg or 40 µg microsomal protein per reaction. The reaction mixture consisted also of a hot/cold steroid mix (15 µM or 150 µM (^14^C)-progesterone (20’000 cpm/rct), 50 µM (^3^H) pregnenolone (40’000 cpm/rct) or 50 µM (^3^H) 17α-hydroxypregnenolone (50’000 cpm/rct), 1 mM NADPH or 1 mM NAD+ in a total volume of 200 µl 50 mM KIP buffer. Microsome assays were performed for 25–120 min at 37°C. Cytochrome b5 (0.5 µg/reaction) was used in the CYP17-17,20 lyase assay. Assays were performed in the presence of 0–20 µM compound C or 0–1 M AICAR. Reactions were stopped by using 1∶1 ethylacetate/isooctane, steroids were extracted and separated by TLC. Kinetic behavior and IC_50_ calculation was determined by nonlinear regression using GraphPad Prism (GraphPad Software, Inc. San Diego, CA).

### Cell Transfection and Dual Luciferase Assay

We used Lipofectamine 2000 (Invitrogen) to either transfect an empty vector (pGL3, Δluc) or promoter-reporter constructs (-3.7CYP17, -1.05HSD3B2, -1.3b5, -325POR, -1.08SULT2A1) into NCI-H295R cells (1.5×10^5^ cells/well in a 24-well format; Falcon 3047; BD Biosciences, Bedford, MA, USA). The transfection mixture per well contained 0.6 µg plasmid, 25 ng Renilla luciferase reporter vector (pRL-TK; Promega), 2 µl Lipofectamine 2000 Reagent (Invitrogen) and 50 µl OptiMEM (GIBCO). Briefly, cells were transfected in suspension for 5 hours, then cells were washed and normal growth medium was added overnight. After 24 hours, cells were treated in serum-free medium with 1 mM AICAR or 20 µM compound C for 48 h. Following this, cells were lysed and assayed for luciferase activity using the dual luciferase reporter assay system according to manufacturer (Promega).

### Respirometric Measurements

We used the OROBOROS® oxygraph, a two chamber respirometer equipped with a peltier thermostat and integrated electromagnetic stirrers. The oxygen concentration was recorded using the software DatLab (OROBOROS® instruments) and oxygen consumption rates were calculated and expressed as specific oxygen consumption rates (pmol O_2_/s/10^6^ cells). Measurements were performed using 0.5–1.5×10^6^ cells/ml in 2 ml of respiration buffer Mir05 [21] at 37°C with continuous stirring. The following substrate-uncoupler-inhibitor titration regime was applied. After measuring the routine respiration (no additions), the cell membrane was permeabilised with 8.1 mM digitonin (1 µl/10^6^ cells). Complex I dependent respiration was measured with malate (2 mM) and pyruvate (5 mM) after addition of ADP (2 mM). Cytochrome C (10 µM) was added to test for the integrity of the outer mitochondrial membrane. The maximal coupled respiration of complexes I and II was stimulated by the addition of succinate (10 mM). By uncoupling with FCCP the maximum capacity of the electron transport system was obtained. Inhibition with rotenone (0.5 µM) shows the maximal uncoupled respiration via complex II. Complex III was inhibited with antimycin A (2.5 µM) to estimate the residual oxygen consumption.

### Mitochondrial Isolation and Respiratory Chain Analysis

Isolation of mitochondria and activity measurements of citrate synthase (CS), NADH coenzyme Q reductase (CI), succinate dehydrogenase (CII), ubiquinol-cytochrome c reductase (CIII), cytochrome c oxidase (CIV) and MgATPase (CV) were determined spectrophotometrically as previously described [22].

### Data Analysis

Statistical analyses were performed using the statistical software Prism 4 (GraphPad Software, Inc. San Diego, CA). Statistical differences between mean values were evaluated by unpaired student's t test or one-way ANOVA followed by the Bonferroni posttest where appropriate. Quantitative data represent the mean of at least two independent experiments, error bars are indicated as the mean ±SEM or ±SD. Significance was set at * *P*<0.05, ** *P*<0.01 and *** *P*<0.001.

## Results

### Steroidogenic Cells and Tissues Express all Subunits of the AMP-activated Kinase

**Figure 1:**
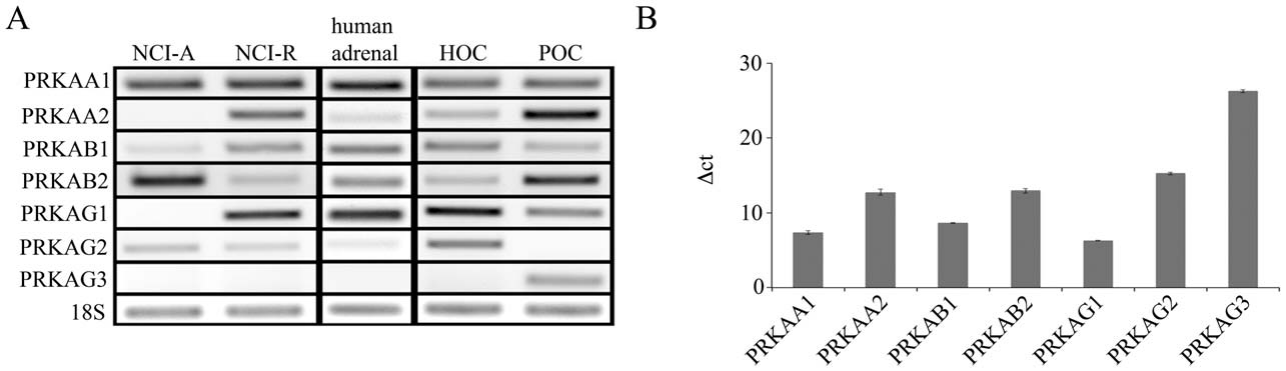
Expression pattern of the subunits of AMPK (PRKA) in steroidogenic tissues. RNA was extracted from human adrenal NCI-H295 cells, adult human adrenal tissue and human as well as porcine primary ovarian cell culture. A, semiquantitative RT-PCR (30 cycles) was performed on 100 ng of reverse-transcribed total RNA using specific primers and PCR products were separated on a 1.5% agarose gel and visualized with ethidium bromide. Shown is a representative gel of three independent experiments. B, QRT-PCR was performed on 50 ng cDNA obtained from NCI-H295R cells using ABsolute QPCR SYBR Green Mix. Data represent calculations of two independent experiments where 18S rRNA served as internal control.

To assess the role of AMPK in steroidogenic tissues, we studied the expression profile of AMPK genes (*PRKA*) by RT-PCR. We detected at least one isoform of each subunit in human adrenal NCI-H295A and NCI-H295R cells, primary cell cultures of human and pig ovaries as well as human adrenal tissue (Figure 1A). Although both NCI-H295A and NCI-H295R cell lines derive from the same adrenocortical tumor [23], they showed a different expression pattern of AMPK subunits. Similarly, we recently reported differences in their steroid profile [24]. The profile of AMPK subunits of human adrenal tissue and human and porcine primary ovarian cell cultures rather resembled the profile of NCI-H295R cells (Figure 1). The subunit AMPKγ3 (gene: *PRKAG3*) was exclusively present in primary pig ovary cells but not in human steroid cells or tissue. In humans, AMPKγ3 seems predominantly to be expressed in skeletal muscle [25].

Since we were particularly interested in NCI-H295R cells for our further studies, we also determined the expression profile of all subunits of AMPK by qRT-PCR in this cell line. The qRT-PCR confirmed the expression pattern for all subunits of AMPK (Figure 1B).

### Overexpression of Wild-type/Dominant Negative AMPKα and Attempted Knockdown (shRNA) in NCI-H295R Cells

**Figure 2:**
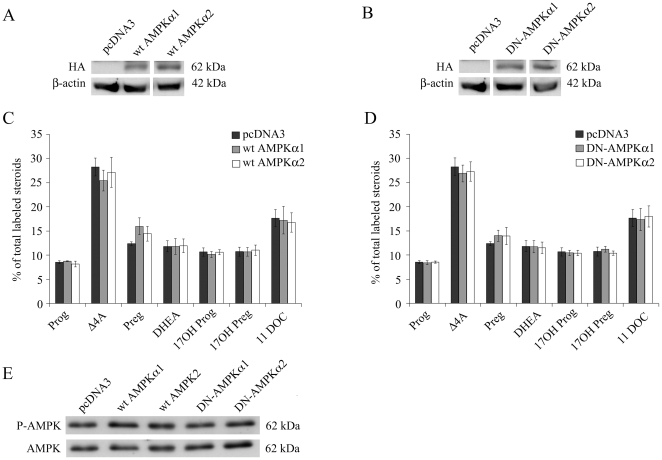
Effect of AMPK overexpression on steroidogenesis in NCI-H295R cells. Wild-type (wt) and dominant negative (DN) subunits of AMPKα1 and AMPKα2 were transiently overexpressed in NCI-H295R cells using specific expression vectors. Steroid production was analyzed by thin layer chromatography (TLC) using radiolabeled (^3^H) pregnenolone (100,000 cpm/35-mm well) as precursor. A–B, representative Western blots showing expression of HA-tagged protein of AMPKα1 or AMPKα2. β-actin served as a loading control. C–D, steroid profiles of transfected NCI-H295R cells showing no effect of AMPK overexpression (C) and no effect of dominant negative AMPKα1 or AMPKα2 (D). Quantification of steroids on TLC was performed from two independent experiments and is given as mean±SEM. Δ4A, androstenedione; Preg, pregnenolone; 17OH-Prog, 17α-hydroxy-progesterone; 17OH-Preg, 17α-hydroxypregnenolone; DHEA, dehydroepiandrosterone; 11 DOC, 11-deoxycortisol. E, Western blot showing AMPK-phosphorylation upon overexpression or blocking of alpha 1 and 2 subunits of AMPK.

We studied the role of AMPK on steroidogenesis by overexpressing wild-type or dominant negative AMPKα1/2. For that NCI-H295R cells were transfected with specific AMPKα constructs which were previously successfully used in studies on the regulation of tuberous sclerosis complexes that include phosphorylation of AMPK [18]. Overexpressing these wild-type or dominant negative α-catalytic subunits 1 and 2 in NCI-H295R cells (Figure 2A, B), we found no effect on steroid biosynthesis (Figure 2C, D), but also no effect on AMPK phosphorylation (Figure 2E) indicating that these subunits may not be of importance in this cells, or that the cellular background may be too strong to be manipulated, or that counter regulatory mechanisms may be in place. Therefore, we attempted to silence endogenous AMPK expression with different shRNA constructs using various approaches such as transient or stable transfection. However, several trials using transient as well as stable transfection with shRNA constructs failed. Since our NCI-H295R cells did not survive any of the silencing procedures in the requested time frame, we suggest that AMPK is crucial for NCI cell survival and/or metabolism.

### Serum-free Growth Conditions Decrease Phosphorylation But Not Activity of AMPK

**Figure 3:**
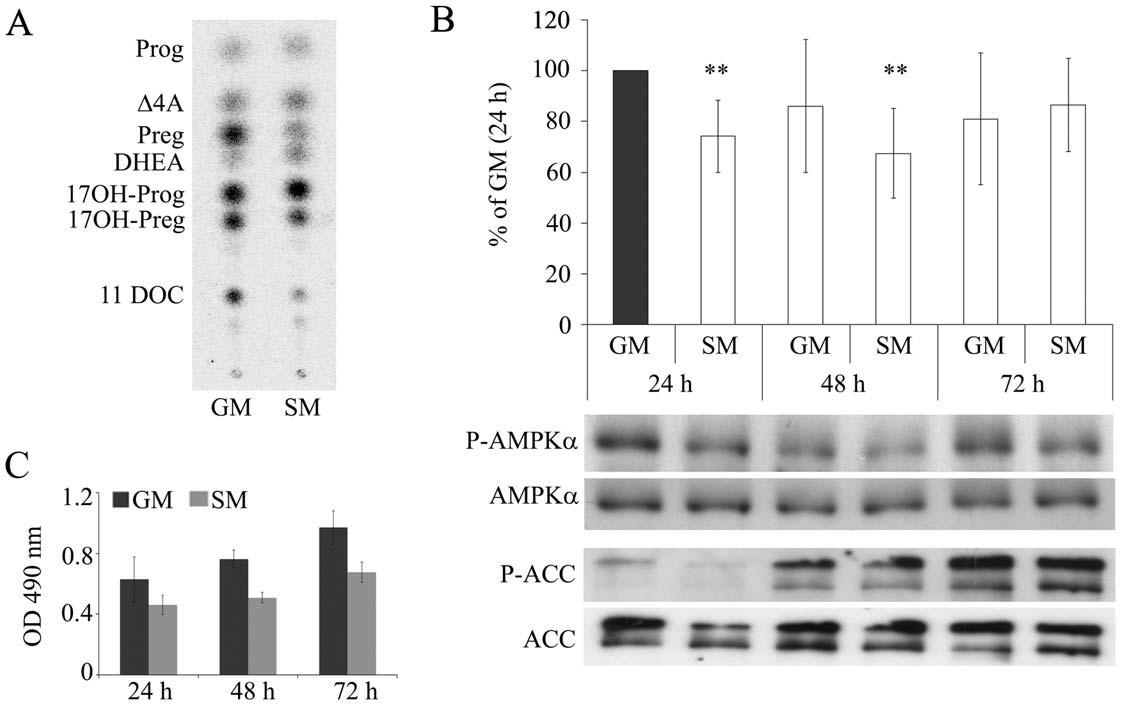
Effect of different growth conditions on phosphorylation of AMPKα and ACC, and on steroidogenesis and cell proliferation of NCI-H295R cells. Cells were grown in regular growth medium (GM) or serum-free medium (SM) for 24–72 hours. Protein extracts were prepared and analyzed by Western blot. Steroids were extracted from medium and resolved by TLC. A, representative TLC analyzing steroid profile of the cells grown under GM and SM conditions after 24 hours. B, Western blot showing diminished phosphorylation of AMPKα in SM after 24 and 48 hours. Quantification was performed on five independent experiments. Results are expressed as a percentage of growth medium (24 h), mean±S.D. **, *P*<0.01. In addition, Western blots were performed for (phospho-)ACC in NCI cells cultured for 24–72 hours in GM or SM. C, cell proliferation was assessed in NCI-H295R cells grown in GM and SM for 24–72 hours using a cell proliferation assay. Results are expressed as OD490 nm, error bars are ±S.E.M of two independent experiments.

Previous studies have shown that starvation growth condition promote androgen production in NCI-H295R cells (Figure 3A) [3]. To assess whether AMPK signaling is involved in starvation changing the steroid profile, we studied phosphorylation of AMPKα in NCI-H295R cells under various growth conditions over time. Therefore, we cultivated NCI-H295R cells in growth medium or serum-free medium for 24, 48 or 72 hours and determined phosphorylation of AMPKα by Western blot. Phosphorylation of AMPKα was decreased in cells grown in serum-free medium for 24 and 48 hours to 74% (±14.2) and 67% (±17.7) respectively (Fig. 3B). By contrast, at the AMPK downstream signaling level, ACC phosphorylation changed over time but showed no difference between NCI cells grown in normal versus serum-free medium, suggesting that starvation does not alter AMPK signaling significantly.

To assess effects of different growth conditions on cell proliferation, we compared proliferation of NCI-H295R cells grown in normal medium (GM) versus starvation medium (SM). It appeared that NCI-H295R cells proliferate slightly faster in GM than SM over 72 hours (Fig. 3C) but the difference was not statistically significant.

### Effect of AMPK on Androgen Production

**Figure 4:**
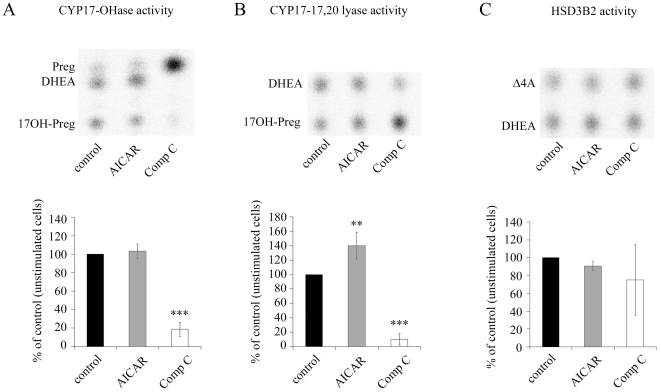
Effect of AMPK activator AICAR and inhibitor compound C on steroidogenesis in NCI-H295R cells. NCI-H295R cells were either stimulated with 1 mM AICAR or inhibited with 20 µM compound C (Comp C) for 48 h. HSD3B2 activity was blocked by 1 µM trilostane to specifically study the CYP17-OHase and CYP17-17,20 lyase activities. HSD3B2 activity was studied as a conversion of DHEA into androstenedione. Steroidogenesis was either labeled using 100,000 cpm/35-mm well of (^3^H) pregnenolone (Preg), (^3^H) 17OH pregnenolone (17OH-Preg) or (^3^H) dehydroepiandrosterone (DHEA) as substrates for 90 min. Steroids were extracted from medium and resolved on TLC plates. A–C, representative TLCs (upper panel) and quantifications (lower panel) of CYP17-OHase (A), CYP17-17,20 lyase (B) and HSD3B2 (C) activities. Quantification was performed on four independent experiments. Results are expressed as a percentage of control, error bars are ±S.D. Δ4A, androstenedione. **, *P*<0.01; ***, *P*<0.001.

To assess the effect of AMPK on androgen production, we determined steroid profiles by thin-layer chromatography of NCI-H295R cells treated with the AMPK activator 5-aminoimidazole-4-carboxyamide ribonucleoside (AICAR; 1 mM) and with the inhibitor compound C (6-(4-(2-Piperidin-1-yl-ethoxy)-phenyl))-3-pyridin-4-yl-pyrrazolo(1,5-a)-pyrimidine; 20 µM), also known as dorsomorphin. CYP17-OHase activity was tested with radiolabeled pregnenolone, 17,20 lyase with 17OH-pregnenolone as substrates respectively in presence of trilostane for blocking HSD3B2 activity. HSD3B2 activity was tested with radiolabeled DHEA substrate. This analysis revealed that both, the activity of CYP17-OHase (Fig. 4A) and CYP17-17,20 lyase (Fig. 4B) were inhibited by compound C, whereas only CYP17-17,20 lyase activity was increased by AICAR (Fig. 4B). The activity of HSD3B2 was neither altered by AICAR nor compound C after 48 h of treatment (Fig. 4C).

### Effect of AICAR and Compound C on AMPK Phosphorylation and CYP17 Expression

**Figure 5:**
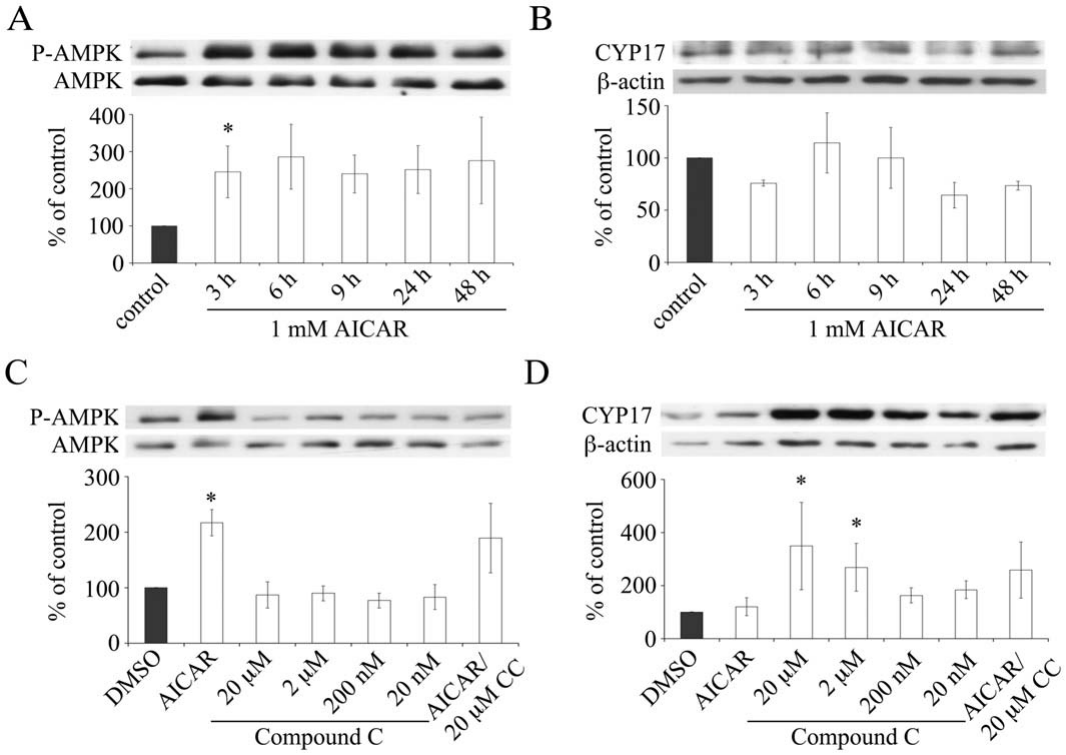
Effect of AICAR and compound C on phosphorylation of AMPK and on CYP17 expression in NCI-H295R cells. A–D, cells were treated and protein extracts were prepared and analyzed by Western blot. A–B, NCI-H295R cells were stimulated with 1 mM AICAR for 3–48 h. An increase in AMPK phosphorylation was noted after 3 hours (A) while CYP17 expression remained unchanged (B). C–D, NCI-H295R cells were treated with 20 nM–20 µM compound C (Comp C), 1 mM AICAR or with both for 48 h. Note that compound C attenuated AICAR stimulated AMPK phosphorylation (C) and increased CYP17 expression at higher concentrations (D). Representative Western blots of two to three independent experiments are shown. β-actin served as loading control. Quantitative results are given as mean ± S.E.M.

We demonstrated the effect of AICAR and compound C on phosphorylation of AMPK and the expression of CYP17 in NCI-H295 cells by Western blot. The AMPK activator AICAR is a cell-permeable adenosine analogue which is intracellular converted by adenosine kinase to 5-aminoimidazole-4-carboxamide-1-D-ribofuranosyl-5′-monophosphate (ZMP) and mimics the effect of AMP [26]. Phosphorylation of AMPKα is increased in response to AICAR treatment in several cells like skeletal muscles, ovary granulosa cells and adipocytes [17], [27], [28]. We investigated the effect of AICAR on AMPK phosphorylation in NCI-H295R cells. In literature, AICAR is usually used at a concentration of 1 mM. In NCI cells, AICAR was found to phosphorylate AMPK starting at concentrations of 0.5 mM with a clear dose-effect up to 2 mM (data not shown). Time-course experiment revealed an increased phosphorylation of AMPK in response to 1 mM AICAR within 3 h which remained sustained for 48 h (Fig. 5A). Because of the effects of AICAR and compound C on the activities of CYP17 (Figure 4A, B), we also assessed their impact on CYP17 expression; however, AICAR did not change the expression of CYP17 (Fig. 5B). By contrast, compound C had an inhibitory effect on AMPK phosphorylation (Fig. 5C). Unexpectedly, compound C increased the expression of CYP17 dose-dependent after 48 hours (Figure 5D), suggesting AMPK independent action(s).

### Compound C but not AICAR Affects CYP17 Enzyme Activity Directly

**Figure 6:**
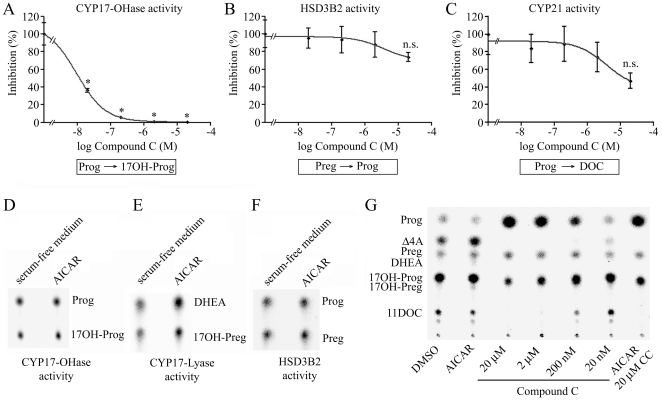
Direct effects of AICAR and compound C on enzyme activities of CYP17, HSD3B2 and CYP21A2 and effect on overall steroidogenesis in NCI-H295R cells. A–F yeast microsomes either co-expressing human CYP17 or CYP21 with human P450 oxidoreductase or expressing human HSD3B2 were incubated with 15 µM or 150 µM (^14^C) progesterone (Prog), 50 µM (^3^H) pregnenolone (Preg) or 50 µM (^3^H) 17α-hydroxypregnenolone (17OH-Preg). At the same time, microsomes were incubated with either various concentrations of compound C or 1 mM AICAR to assess their effect on the activities of CYP17-OHase (A, D), CYP17-17,20 lyase (E), HSD3B2 (B, F) and CYP21A2 (C). G, effect of AICAR and compound C on overall steroidogenesis in NCI-H295R cells. Experiments were repeated independently two to three times. Error bars represent ±SEM. *, *P*<0.05.

Several papers describe AMPK-independent effects of AICAR and compound C in various cells such as a gliogenic effect of AICAR by activating the JAK/STAT3 pathway in neuronal stem cells of rats or the inhibition of 3T3-L1 preadipocytes by compound C as a result of increased p21 content [29], [30], [31], [32]. To exclude or establish direct effects of the chemicals AICAR and compound C on steroidogenic enzymes, we performed *in vitro* enzyme kinetic assays using recombinant proteins from yeast microsomes [33]. We found a direct dose-dependent inhibition of the CYP17-OHase activity by compound C with a calculated IC50 of 9.6 nM (Fig. 6A). By contrast, compound C did not directly affect the activities of HSD3B2 and CYP21A2 (Fig. 6B, C). Furthermore, AICAR did not change the activities of CYP17A1 (-OHase and 17,20 lyase) and HSD3B2 (Fig. 6D–F). These same results were then generated in NCI-H295R cells treated with AICAR and compound C assessing their steroid profile (Fig. 6G). In contrast to the enzyme kinetic assays, AICAR slightly enhanced androgen production in the cell model (Fig. 6G+Fig. 4B), suggesting involvement of AMPK signaling. Compound C, which already inhibited CYP17 activity directly, inhibited the conversion of pregnenolone to CYP17-dependent intermediates such as 17OH derivates (17OH-pregnenolone and 17OH-progesterone) and androstenedione in a dose-dependent fashion (Figure 6G). These data indicate that compound C is a direct inhibitor of CYP17.

### Cell proliferation of NCI-H295R Cells Treated with AICAR or compound C

**Figure 7:**
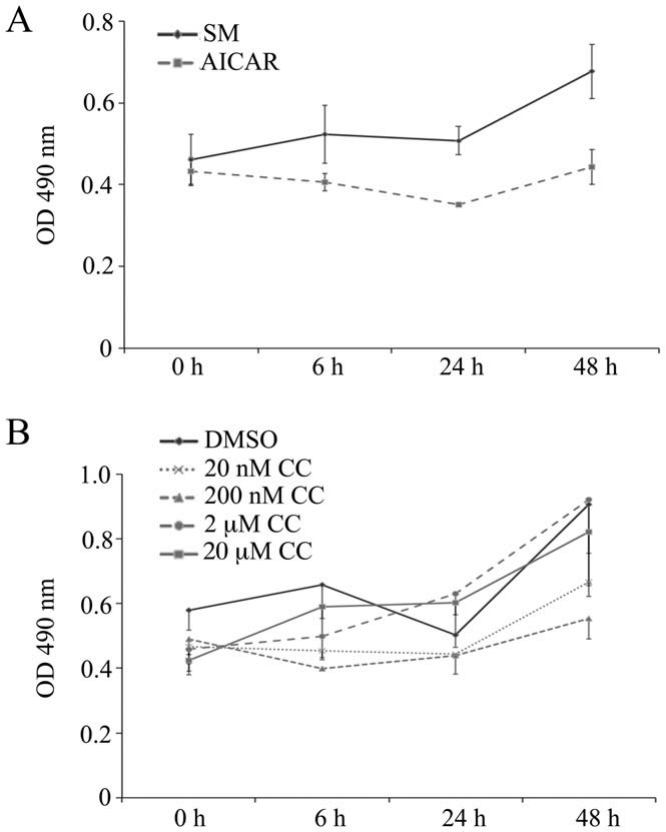
Cell proliferation of NCI-H295R cells. Cells were treated with 1 mM AICAR (A) or 0–20 µM compound C (B) in serum-free medium for 0–48 hours. A commercially available cell proliferation assay was performed (Promega). Results are expressed as OD490 nm which corresponds directly to cell viability in this assay. Data are the mean of two independent experiments ± S.E.M.

We performed cell proliferation assays of NCI-H295R treated with 1 mM AICAR or 0–20 µM compound C for 0–48 h to study their effect on cell viability. We found that AICAR slowed down cell proliferation over 72 hours insignificantly (Fig. 7A), while compound C had no effect at any concentration tested (Fig. 7B).

### Effect of AICAR and Compound C on Promoters of Genes Involved in Androgen Biosynthesis

**Figure 8:**
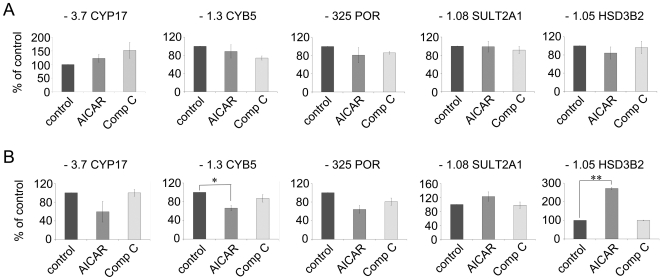
Studies of the effect of AICAR and compound C on promoter activities of genes involved in androgen production. NCI-H295R cells were either transfected with an empty vector (pGL3, Δluc) or promoter luciferase reporter constructs ( -3.7 CYP17, -1.05 HSD3B2, -1.3 b5, -325 POR, -1.08 SULT2A1) and subsequently treated with 1 mM AICAR or 20 µM compound C for 6 h (A) or 48 h (B). Following transfection and treatment, promoter activities were assessed by Dual luciferase assay readout (Promega). Quantification data represent two independent experiments performed in duplicates. Results are expressed as a percentage of control, mean ±S.E.M. *, *P*<0.05; **, *P*<0.01.

To test whether AICAR or compound C modulate the activities of promoters of steroidogenic genes, we transfected NCI-H295R cells with specific promoter constructs (-3.7 CYP17, -1.05 HSD3B2, -1.3 CYb5, -325 POR, -1.08 SULT2A1). We treated the transfected cells with 1 mM AICAR or 20 µM compound C for 6 h and 48 h and measured the specific promoter activities by the dual luciferase assay system (Promega). Our data showed that neither AICAR nor compound C influenced the activities of the CYP17, POR or SULT2A1 promoters after 6 or 48 hours (Fig. 8). Moreover, compound C did not change the activities of CYB5 and HSD3B2 (Fig. 8). By contrast AICAR was found to enhance the activity of the HSD3B2 promoter after 48 hours (Fig. 8), but this activation obviously did not result in an increase in enzyme activity (Fig. 4C). AICAR slightly decreased the activity of the CYB5 promoter. As CYB5 is known to enhance the CYP17-17,20-lyase activity [20], one would expect that a decrease in CYB5 activity would prompt a reduction in CYP17-17,20 lyase activity; however, as shown in Figure 4B, AICAR increased the CYP17-17,20 lyase activity significantly suggesting stronger regulation at the posttranscriptional level.

### Mitochondrial Function of NCI-H295R Cells Grown under Starvation Conditions

The respiratory chain in the mitochondria plays a pivotal role for the cellular energy homeostasis and thus the intracellular AMP∶ATP ratio which can regulate phosphorylation of the AMPK. Therefore, to assess whether starvation affects AMPK activity indirectly, we investigated mitochondrial function in NCI-H295R cells. NCI-H295R cells were cultivated in growth medium and starvation medium for 48 h and then analyzed by high-resolution respirometry using a substrate uncoupler inhibitor titration (SUIT) protocol as described [34] and by spectrophotometric measurements of the individual OXPHOS complexes [22].

**Figure 9:**
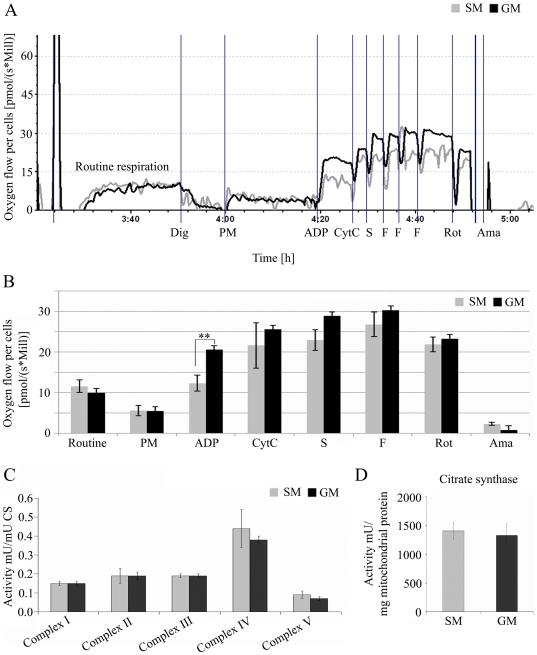
Energy state assessed by mitochondrial function in starved NCI-H295R cells. A,B. Oxygen consumption was assessed by high-resolution respirometry in digitonin-permeabilized NCI-H295R cells grown in growth medium (GM) versus starvation medium (SM). A, representative oxygraph traces. B, quantitative analysis of oxygen consumption is given as O_2_ flow per cells in pmol/(s*Mill.). The addition of substrates, uncoupler and inhibitors is indicated on the x-axis. Routine: no addition (control); Dig: digitonin; ADP: adenosine diphosphate; PM: pyruvate and malate; CytC: cytochrome c; S: succinate; F: carbonyl cyanide p-trifluoromethoxyphenylhydrazone (FCCP); Rot: rotenone; Ama: antimycin A. Data represent means +/−SD, n = 3. C,D. Spectrophotometric assays in isolated mitochondria. The specific activities of the respiratory chain complexes were obtained as mU per mg mitochondrial protein and are expressed as ratios to citrate synthase (CS) activity serving as quality control of equal mitochondrial content (D). Complex I (NADH:ubiquinone oxidoreductase), complex II (succinate dehydrogenase), complex III (ubiquinol-cytochrome c reductase), complex IV (cytochrome c oxidase), complex V (ATPase). Results are presented as mean of 2–3 independent experiments. Error bars represent +/−SD. **, *P*<0.01.

Routine respiration of the cells grown under different conditions was similar (Figure 9 A, B). But high-resolution respirometry after cell permeabilisation with digitonin (Dig) revealed overall lower O_2_ flux in cells cultivated in starvation medium when compared to cells grown in normal growth medium (Figure 9 A,B). This effect was independent of added substrates indicating that there is no reduction in the activity of a single specific complex of the respiratory chain. A significant difference was measured with pyruvate and malate (PM) in the presence of ADP as substrates for complex I (Figure 9B). By the addition of cytochrome c this difference disappeared suggesting a damaging effect of digitonin on the outer mitochondrial membrane. Furthermore, the individual enzymatic activities of the respiratory chain complexes showed no differences in mitochondria isolated from NCI-H295R cells grown in starvation medium compared to normal growth medium (Figure 9C). Citrate synthase activity as a marker of mitochondrial content (and experimental control) did not differ between NCI-H295R cells grown under different conditions (Figure 9D). Thus, starvation does neither seem to have a specific impact on mitochondrial oxygen consumption nor on the OXPHOS complex activities.

## Discussion

We investigated the role of AMP-activated protein kinase (AMPK) signaling on androgen biosynthesis in human adrenal H295R cells. As CYP17A1 and HSD3B2 genes/proteins are essential for adrenal androgen production, our studies focused on those targets. Overall, we found that AMPK activation enhances androgen production. Specifically, AMPK-activating chemical AICAR increased CYP17-17,20 lyase activity without changing CYP17-OHase activity and HSD3B2 activity. AMPK-inhibiting chemical compound C revealed an inhibition of CYP17A1 activities but not HSD3B2 activity, but was found to be a direct inhibitor of CYP17A1 enzyme and not a specific AMPK inhibitor only. In previous work, we showed that starvation enhances androgen production in NCI-H295R cells; therefore we hypothesized that AMPK signaling which is at the core of energy homeostasis may be the mediator. However, we found that starvation growth conditions lead to dephosphorylation but not decreased activity of AMPK in NCI-H295R cells indicating that AMPK signaling is not mediating the starvation induced androgen production.

Recent studies have shown that all AMPK subunits are expressed in steroidogenic ovarian granulosa cells in rats, hens and bovines [16], [17], [35]. We found expression of at least one isoform of each subunit of AMPK in human adrenal NCI-H295A and NCI-H295R cells, human adrenal tissue as well as primary cell cultures of human and pig ovaries. Although NCI-H295A and NCI-H295R cells originate from the same adrenocortical carcinoma, they show profound differences in their steroid biosynthetic characteristics [24]. Our data now additionally illustrate that the expression profile of AMPK is different between these two cell lines. Interestingly, the expression pattern of AMPK subunits of NCI-H295R cells resembles that of the human adrenal tissue when compared to NCI-H295A cells.

Maintenance of energy balance is crucial for proper function of all organisms and cells at long term. However, once in a while systems get stressed and energy consumption rises. To regain balance, adaptive regulations are necessary to either generate more energy (mostly ATP) and/or to switch to an energy saving mode [5]. AMPK is regarded the mammalian energy sensor protein which switches off ATP-consuming metabolic pathways and switches on ATP-producing catabolic processes in times of metabolic stress and sensed ATP depletion [5], [7]. Starvation is a “stress”. In previous work, we showed that serum-free, starvation conditions which represents stress for a cell, shifts steroidogenesis of NCI-H295R cells towards androgen production [3], but the underlying signaling cascade remains unknown. Thus, the fact that stress through starvation obviously enhances androgen production, prompted us to speculate that AMPK signaling might be involved. Because nutritional stress usually prompts AMPK phosphorylation and activation [5], [7], we expected an increase in AMPK phosphorylation in NCI-H295R cells grown under starvation conditions. However, this was not the case. We found a decrease in the phosphorylation of AMPK but no change in its activity to phosphorylate the downstream signaling molecule ACC in NCI-H295R cells after starvation. Since the respiratory chain of the mitochondria is involved in cellular energy balance contributing to the cellular AMP∶ATP content and since the AMP∶ATP ratio regulates AMPK phosphorylation, we assessed mitochondrial function searching for a possible indirect effect of starvation on AMPK. Oxygraphic measurements revealed that total oxygen consumption of starved cells was similar to normally grown cells for routine respiration. By contrast, after cell permeabilisation with digitonin and specifically adding pyruvate and malate together with ADP, oxygen consumption was found decreased in starved cells, suggesting a possible effect on complex I. However, since this effect was reversed by cytochrome c and since in intact cells no difference in the O_2_ flux was observed, we attribute this effect rather to a damage of the outer mitochondrial membrane through digitonin. Nevertheless this observed cytochrome c effect was more pronounced in starved cells indicating that starvation makes the outer mitochondrial membrane of NCI-H295 cells more prone to damage. Overall, our mitochondrial function studies reveal that starvation does not really change the mitochondrial respiration rate. However, from our studies we cannot exclude that the cellular AMP∶ATP content is modulated by other metabolic pathways influenced by starvation. To solve this question, we may aim at measuring the cellular AMP∶ATP content directly in future studies.

Compound C is sold by biochemical companies as a specific inhibitor of AMPK. However, compound C has been reported to also have AMPK-independent effects [29], [30]. In addition to the described effects, we show novel AMPK-independent actions of compound C on steroidogenesis. We observed a specific, dose-dependent, direct inhibition of the CYP17 enzyme activity through compound C. By contrast, enzyme activities of HSD3B2 and CYP21A2 were not affected by compound C directly. Compound C was also found to increase the expression level of CYP17, although CYP17 activity was nevertheless inhibited. This increase in the amount of CYP17 protein by compound C may be due to some protein stabilization properties of the compound. In fact, Nam et al. showed that compound C increases the amount of p21, a protein which is involved in cell-cycle progression, in preadipocytes [30]. In this study, compound C delayed the decrease of p21 levels in cycloheximide-treated (chemical inhibitor of protein synthesis) cells, indicating that compound C may cause protein stabilization [30]. The importance of our observation for the field of steroidogenesis may be demonstrated in a recent publication, in which compound C was assumed to be a specific inhibitor of AMPK [36]. Authors describe an increase in cortisol secretion after treating NCI-H295R cells with adiponectin. Since adiponectin may act on AMPK signaling and since treatment of NCI-H295R cells with compound C inhibited cortisol secretion, it was concluded that the effect of adiponectin is mediated through AMPK. Knowing that compound C is a very potent, direct CYP17 inhibitor (and that it is essential for cortisol biosynthesis), it may not be used for addressing questions regarding AMPK signaling in steroidogenic cells.

In summary, we investigated the role of AMPK signaling in human adrenal NCI-H295R cells. We show that under starvation growth conditions which enhance androgen production, AMPK phosphorylation but not activity is decreased. We also show that AMPK phosphorylation and activation increases androgen production through enhanced CYP17-17,20 lyase activity. Therefore, we conclude that AMPK signaling can modulate androgen production but that it is not the mediator of the starvation effect on androgen biosynthesis. In addition, we found that compound C (which is on the market as a “specific” AMPK inhibitor) is a potent, direct CYP17 enzyme inhibitor.
